# Dimensions of Warm Parenting Attributions Differentiate Conduct Problem Subtypes in Young Children

**DOI:** 10.1007/s10802-023-01111-7

**Published:** 2023-08-15

**Authors:** Silvana Kaouar, Georgette E. Fleming, Bryan Neo, David J. Hawes, Valsamma Eapen, Eva R. Kimonis

**Affiliations:** 1 Parent-Child Research Clinic, School of Psychology, The University of New South Wales, Sydney, NSW Australia; 2 School of Psychology, The University of Sydney, Sydney, Australia; 3 School of Psychiatry, The University of New South Wales, Sydney, NSW Australia

**Keywords:** Conduct problems, Callous-unemotional traits, Primary variant, Secondary variant, Parenting style

## Abstract

Understanding the developmental psychopathology of child conduct problems (CP) has been advanced by differentiating subtypes based on levels of internalizing problems (INT) and/or callous-unemotional (CU) traits (i.e., low empathy/guilt, poor motivation, shallow/deficient affect). The current study sought to elucidate prior inconsistencies in the role of warm/positive and harsh/negative parenting subcomponents in CP by differentiating subtypes on the basis of INT and CU traits. Parents of 135 young children (*M* age = 4.21 years, *SD* = 1.29) referred to specialty clinics for the treatment of CP completed pre-treatment measures of parenting and rated their child’s levels of CP, INT, and CU traits. Results of planned comparisons revealed that mothers of children classified as secondary CU variants (high CU/ high INT) reported fewer overall warm attributions toward their child, compared with CP-only (low CU) children. They also reported a more negative dyadic relationship characterized by feelings of anger/hostility, active avoidance and/or a desire to do harm to their child relative to primary CU variants (high CU/ low INT). Mothers of primary CU variants attributed fewer good and altruistic intentions towards others in their child, relative to CP-only children. Subtypes were undifferentiated on observed positive and negative parenting behaviors, indicative of a disconnect between parenting behaviors and cognitions for mothers of children high on CU traits. Findings are discussed in relation to their theoretical and practice implications, and in guiding future research.

**Supplementary Information:**

The online version contains supplementary material available at 10.1007/s10802-023-01111-7.

Early-onset conduct problems (CP) are a risk factor for stable and persistent impairment across childhood through to adulthood (Burke et al., 2014; Fergusson et al., 2005; Neo & Kimonis, 2021; Weeks et al., 2016). There are multiple developmental pathways to childhood-onset CP, with the presence of elevated Callous-Unemotional (CU) traits (i.e., low empathy/guilt, shallow affect) identifying individuals with frequent, aggressive, and persistent antisocial behavior (see Frick et al., 2014). While this severe pattern of CP in children with elevated CU traits is thought to be uniquely underpinned by dispositional differences in neurocognition, psychophysiology, socioemotional sensitivity, and genetic vulnerability (Viding & Kimonis, 2018), parenting behaviors are also important in the etiology of CU traits and associated CP (Waller, Hyde et al., 2018).

Parenting factors are central to developmental models of CP, with strong and causal relationships for harsh and coercive parenting specifically (Patterson et al., 1989; Viding et al., 2009). However, the relative influence of specific subcomponents of parenting on CP appears to vary according to level of CU traits. Several studies find that harsh and coercive parenting is less associated with the CP of school-age children and adolescents with elevated CU traits, relative to those low on CU traits (Wootton et al., 1997; Pasalich et al., 2011a; cf. Waller et al., 2015a). Instead, low parental warmth is more strongly associated with CP when CU traits are high versus low (Kochanska et al., 2013; Kroneman et al., 2011). For example, global parental warm attributions, coded from parents’ five-minute speech samples (FMSS; Magaña et al., 1986), was significantly negatively correlated with CP at moderate and high levels of CU traits, but not low levels among a sample of 4-12-year-old clinic-referred boys (Pasalich et al., 2011a). While Waller et al. (2015a) also found that warm parental attributions coded from FMSS were more strongly negatively associated with CP for toddlers with elevated CU traits, they did not find evidence for a moderating influence of CU traits on the association between CP and observed parental harshness.

One possible explanation for these mixed findings is that harsh parenting is relevant to the development of CP for only a subset of children with elevated CU traits, namely those with ‘secondary’ CU traits who tend to experience extreme high levels of harsh, coercive, and abusive parenting (for a review see Craig et al., 2021b). Relative to primary CU variants who are temperamentally fearless and emotionally hypo-reactive to negative stimuli (Kimonis et al., 2012; Craig et al., 2021b), secondary CU variants are characterized by high internalizing symptoms and heightened emotional sensitivity and dysregulation (Fanti & Kimonis, 2017; Ezpeleta et al., 2017; Craig & Moretti, 2019). These characteristics likely increase their susceptibility to problematic outcomes resulting from their negative parenting experiences. Differences between primary and secondary CU variants are evident from early childhood through to adulthood (Ezpeleta et al., 2017; Fanti & Kimonis, 2017; Kimonis et al., 2017; Sethi et al., 2018). However, research on CU variants in young children and particularly within clinical samples is scarce, which is a knowledge gap the present study seeks to fill.

Although primary and secondary CU variants are phenotypically indistinguishable in their levels of CU traits, they are theorized to differ in their etiology. Developmental models specify that the origins of primary CU traits are rooted in biological and temperamental risk factors, whereas secondary CU traits develop following adverse and traumatic experiences in early life, such as parental maltreatment (Craig & Moretti, 2019; Cecil et al., 2014; Kimonis et al., 2011). Putative differences between CU variants in biological, temperamental, and environmental risk have received empirical support. For example, youth classified as primary CU variants showed significantly lower heart rates (Fanti & Kimonis, 2017) and greater deficits in emotional attention to distress stimuli on a dot-probe task (Kimonis et al., 2012), compared with secondary CU variants who had higher heart rates and were hypervigilant to negative stimuli. Only two prior studies to our knowledge have investigated risk factors differentiating primary and secondary CU variants in samples including young children, providing preliminary support for the existence of CU variants in early childhood (Dadds et al., 2018; Ezpeleta et al., 2017). The present study extends these findings by investigating CU variants in the largest sample to date of exclusively clinic-referred young children, thus advancing knowledge about the developmental pathways to CP and primary and secondary variants of CU traits.

Given the central role of maltreatment to developmental models of secondary CU traits, it stands to reason that the relative influence of parental harshness and warmth in the development of CU traits may differ between CU variants. Several studies support a relationship between secondary CU traits and harsh and coercive parenting in both school-age children (Goulter et al., 2017; cf. Bégin et al., 2021) and adolescents (Craig et al., 2021a; Flexon, 2015; Kimonis et al., 2011; Waller et al., 2018a). For example, Goulter et al. (2017) found that high-anxious secondary CU variants experienced greater parent- and child-reported harsh, negative, and inconsistent parenting at age 7, relative to primary CU variants and a low problems control group. However, not all studies are consistent in finding that secondary CU variants receive more harsh parenting than other CP subtypes. For instance, a longitudinal study of young children found that secondary CU variants did not differ from primary CU variants or controls on parent-reported punitive or positive parenting at age 3, but did experience greater parental inconsistency and lower limit-setting than control children (Ezpeleta et al., 2017). Thus, while many studies support a positive association between secondary CU traits and harsh/negative parenting, there is some inconsistency in finding that parenting factors differentially relate to CU variant groups.

However, there are two important limitations to literature on parenting and CU variants that may explain previous null findings in variant-specific differences in parenting factors. First, few studies use clinic-referred samples of children and compare against a group of children with CP without CU traits (i.e., ‘CP-only’). Instead, most prior studies compare variants to a low-problems (i.e., low CP/low CU) control group (e.g., Craig et al., 2021a; Humayun et al., 2014; Meehan et al., 2017). While beneficial for understanding deviations from typical development, this approach limits understanding of risk factors relevant to different developmental pathways to CP.

A second limitation of the prior research is its broad, dichotomous examination of positive and negative parenting styles. This literature typically fails to differentiate specific parenting subcomponents within these broader parenting dimensions that are uniquely important to trajectories of CU traits. The limited consideration of nuances in warm and harsh parenting relevant to specific subgroups of children with CP is reflected in prior approaches to measuring parenting dimensions, which typically relied on either rater-based measures or global ratings of observed parenting behaviors. Elucidating specific parenting behaviors and attributions that distinguish subtypes of children with clinically significant CP is vital to understanding their distinct developmental pathways and has potential for informing tailoring of early intervention programs. This is an important endeavor considering that the CP of children with elevated CU traits typically fail to normalize from the best available parenting interventions, which may be due to these interventions not traditionally addressing the distinct, nuanced mechanisms underlying the CP of children with CU traits (Wilkinson et al., 2016).

Recent efforts to adapt parenting interventions to target the unique deficits of young children with elevated CU traits show some promise for improving their treatment outcomes (Fleming et al., 2022; Kimonis et al., 2019). To date, however, little to no consideration has been given to whether CU variant groups require nuanced treatment approaches, despite findings that secondary CU variants are at greatest risk for psychiatric illness, violence, and co-occurring substance use problems (Craig et al., 2021b). To our knowledge, there has been no published research into targeted interventions for children identified as primary or secondary CU variants. However, early intervention for this population is critical, given evidence of the temporal stability of CU traits, which are identifiable from a young age. For example, research supports that CU traits can be reliably and validly measured from age 3 years (Kimonis et al., 2016). These CU traits are distinguishable from other relevant constructs including physical aggression, oppositionality, and attention-deficit/hyperactivity disorder-related behaviors (Waller et al., 2015b, 2017; Wright et al., 2021; Willoughby et al., 2011, 2014), show moderate temporal stability at ages 2 and 3, (Flom & Saudino, 2017), and sex-invariance from age 2.5 to 5 years (Wright et al., 2021). There is accumulating support for early interventions targeted towards young children with elevated CU traits, and research into variant-specific differences in parenting is a possible focal point for these interventions.

## The Current Study

The present study aimed to address the knowledge gap on whether CP subtypes require nuanced treatment approaches, while overcoming limitations of past research investigating parenting factors for this population of children. We addressed this goal by examining both global dimensions of parenting factors, and specific empirically- and/or theoretically-determined parenting subcomponents that may differentiate clinic-referred young children with CP disaggregated into CP-only (i.e., low levels of CU traits), primary CU variant (low internalizing), and secondary CU variant (high internalizing) groups. This research is expected to advance understanding of parental influences on different developmental pathways to CP and CU by integrating tests of these key parenting processes in three novel ways. First, we examined specific subcomponents within the broad positive and negative dimensions of parenting factors. This was enabled by using micro-observational coding methods that are capable of capturing parental behaviors at a granular level. Prior research has largely neglected this approach in favor of less resource-intensive macro-observational coding systems, despite its potential for uncovering unique parenting correlates of CP subtypes (Bank et al., 1990).

Second, we examined parental attributions regarding their child’s dispositional traits, the intentions driving their child’s antisocial behaviors, and the quality of the parent-child relationship. Research suggests that parents’ cognitions about their child may influence parent-child interactions. Studies have found that parents who hold child-referent attributions of their child’s behavior (e.g., that misbehaviors are intentional) show greater hostile, coercive, less warm parenting (Bolton et al., 2003) and more reactive discipline that is characterized by anger (Slep & O’Leary, 1998) than parents low on these child-referent attributions. A recent study examining parental attributions of school-aged children with CP and varying levels of CU traits found that child-referent cognitions regarding the intentionality/controllability of their child’s behaviors were associated with more negative parenting when CU traits were high, but more positive parenting when CU traits were low (Arslan et al., 2022). While other research suggests that mothers of young children with CP and CU traits expressed more negative attributions and fewer positive attributions about their child’s traits and the permanence and intentionality of their child’s CP relative to mothers of children with CP-only (Sawrikar et al., 2019), no study has examined CU variant-related differences in parental attributions. Further, we would expect that greater negative and fewer positive attributions about their child would impact parents’ perceptions and experience of the parent-child relationship.

Third, we assessed global parental warmth/positive parenting factors and harshness/negative parenting factors using multiple methods, namely both macro-social (in the form of parent attributions of their child from speech samples) and micro-observational measures of parenting (in the form of observed parenting behaviors). This multi-method approach is important given prior research with young children found that both observed and expressed attributions of parental warmth predicted the emergence of CU behaviors one year later, independent of CP, and that CU traits differentially impacted future levels of these warm parenting factors (Waller et al., 2014).

### Aims and Hypotheses

The first aim of the current study was to test whether CP subtypes differ on warm/positive parenting factors across both expressed and observed indices. We hypothesized that children with elevated CU traits would have lower expressed and observed parental warmth, assessed globally and across warmth subcomponents, than the CP-only group. We further predicted that these indices of warm parenting would be lowest for children classified as secondary CU variants relative to primary CU variants (Craig et al., 2021b) and CP-only (Fanti & Kimonis, 2017). Specifically, we hypothesized that (1) parents’ warm and positive attributions about their child’s dispositional traits, intentionality, and their feelings of love/care towards their child, and (2) positive parenting behaviors including labeled/unlabeled praise, behavior descriptions, and reflections would be lowest for both CU variant groups relative to the CP-only group, and lower for the secondary CU group compared with the primary CU group.

The second aim of the current study was to test whether CP subtypes differ on harsh/negative parenting factors across both expressed and observed parenting indices. We hypothesized that expressed and observed parental harshness and negative parenting, assessed globally and across specific subcomponents, would be greatest for secondary CU variants, relative to the other CP subtypes, with the lowest parental harshness for primary CU variants, consistent with literature showing greater harsh parenting in association with CP when CU traits are low (Pasalich et al., 2011a), and greatest maltreatment levels for secondary CU variants (Goulter et al., 2017). Specifically, we hypothesized that (1) parental negative attributions about their child’s traits, intentionality, and their relationship with their child, and (2) negative parenting behaviors including negative talk, questions, and commands during free-play, would be highest for secondary CU variants followed by CP-only children, with the lowest levels for primary CU variants, consistent with Goulter et al. (2017) and Pasalich et al. (2011a).

## Method

### Participants

The University of New South Wales (UNSW) and South-Western Sydney Local Health District Human Research Ethics Committees approved all study procedures. Written informed consent was obtained from all participating parents upon arrival for their baseline, initial assessment. Participants were drawn from research studies across two clinics providing early assessment and intervention for children with conduct problems (*N* = 135), one of which oversampled children with elevated CU traits for research trials testing a targeted treatment for this subpopulation (Fleming et al., 2022). Oversampling of children with CU traits was achieved by advertising help for families in managing their preschooler’s difficult behaviors, including temper tantrums, disobedience, anger & irritability, low motivation, little remorse, little empathy, shallow emotions, and where discipline is ineffective. All children presented with conduct problems, although they varied on level of CU traits. From our original sample of 200 children, 135 had mothers who completed all questionnaire study measures, of which a subset of 105 mothers further completed all observed parenting measures. See Supplemental Statistical Materials for analyses comparing groups of children based on whether their mothers had complete or incomplete measures, child age, and sex, across all main study variables. All fathers were invited to participate, where applicable, but only 38 participated in the assessment. The final participant sample included young children (*N* = 135, 77% male, 23% female) between the ages of 2- and 7-years-old (*M* age = 4.21, *SD* = 1.29 years) and their parent(s). The current study used baseline assessment data collected prior to families completing a parent management training intervention.

Children were classified into three CP subtypes according to scores on the 24-item *Inventory of Callous-Unemotional Traits – Preschool Version* (ICU; Kimonis et al., 2016) and the *Achenbach System of Empirically Based Assessment* (ASEBA; Achenbach and Rescorla, 2000, 2001) Internalizing composite scale score. Due to a lack of agreed-upon cut-off score constituting elevated levels of CU traits in young children, a median-split approach was used to categorize children into CP subgroups for this study. When benchmarked within a normative range of scores for a community sample, our cut-off score of 29 was found to equate to approximately 1 standard deviation above the 24-item mean reported for typically developing pre-schoolers (Ezpeleta et al., 2013). This corresponds to an average item rating of 1.20, which falls between ‘Somewhat true’ and ‘Very true’. The majority of past research examining CU variants has been conducted within juvenile justice or community settings. This ICU cut-off score of 29 was also used to identify elevated levels of CU traits in adolescent populations, as determined through both empirical and normative cut-off methods (Colins et al., 2018; Kemp et al., 2021). Using this group-based approach allows us the unique opportunity to examine clinically meaningful CP subgroups within a clinical sample of young children with CP. It also enables others to adopt a consistent approach to studying CU variants within other clinical populations of young children.

Final group classifications for the present study were as follows: CP-only (*n* = 59, scoring below the median split value of 29 on the ICU), primary CU variant (*n* = 38, scoring ≥ 29 on the ICU, < 64*T* cut-off score on CBCL Internalizing), and secondary CU variant (*n* = 38, scoring ≥ 29 on ICU, ≥ 64*T* on CBCL Internalizing).

Planned comparisons revealed no sex differences, *F*(2, 132) = 0.05, *p* = .948, η_p_^2^ = 0.00, or age, *F*(2, 132) = 1.80, *p* = .169, η_p_^2^ = 0.03, between the three CP subtypes. CP scores were highest for secondary CU variants compared to primary CU variants, *F*(1, 132) = 5.10, *p* = .026, η_p_^2^ = 0.04, and the CP-only group *F*(1, 132) = 28.02, *p* < .001, η_p_^2^ = 0.18, and higher for primary CU variants than the CP-only group, *F*(1, 132) = 7.86, *p* = .006, η_p_^2^ = 0.06. Holding CP constant, CU trait scores were significantly higher for the primary CU variant, *F*(1, 131) = 123.82, *p* < .001, η_p_^2^ = 0.49, and secondary CU variant, *F*(1, 131) = 149.82, *p* < .001, η_p_^2^ = 0.53 compared to the CP-only group. Primary and secondary CU variants were undifferentiated in CU trait scores. Lastly, holding CP constant, internalizing scores were significantly higher for secondary CU variants than primary CU variants, *F*(1, 131) = 44.01, *p* < .001, η_p_^2^ = 0.25, and CP-only group, *F*(1, 131) = 26.76, *p* < .001, η_p_^2^ = 0.17. Primary CU variants and the CP-only group did not differ in internalizing scores.

### Procedure

Parents completed questionnaires measuring child CP, CU traits, and internalizing symptoms via hard copy or online Qualtrics survey software as part of a baseline assessment, which was used to assess their eligibility for participation in a clinical research trial for one of the two clinics. During the in-person component of the baseline assessment, parents were invited to complete a FMSS, where they spoke alone for 5-minutes into an audio recorder about “what kind of a person [child] is, and how the two of you get along together”, with no further prompts or interruptions from the assessor. Parents were also invited to complete a dyadic parent-child interaction task with their child, with verbal instructions for this task provided to parents via a wireless earpiece from the assessor observing behind a one-way mirror. Parents completed several other measures that were not a focus of the current study.

### Measures

See Supplemental Methodological Materials for further details of measures used in the present study.

#### Conduct Problems

Child conduct problems were assessed using the intensity scale of the 36-item *Eyberg Child Behavior Inventory* (ECBI; Eyberg and Pincus, 1999). In the present study, mother-reported ECBI Intensity scores demonstrated excellent internal consistency (α = 0.90).

#### CU Traits

CU traits were assessed using 24-item total scores from the preschool version of the ICU (e.g., “Does not care who he/she hurts to get what he/she wants”; Kimonis et al., 2016). In the present study, total mother-reported ICU scores showed good internal consistency (Cronbach’s α = 0.82).

#### Internalizing Symptoms

Child internalizing symptoms were assessed using mother-reported *T*-scores on the 36-item Internalizing composite scale (e.g., “too fearful or anxious”) of the ASEBA (Achenbach & Rescorla, 2000, 2001), as supported by past research using a measure of anxiety and/or broad internalizing scales to differentiate primary and secondary CU variants (Craig et al., 2021b). In the present study, the Internalizing composite scale had good internal consistency (Cronbach’s α = 0.84).

#### Expressed Parental Criticism and Warmth

Parents were asked to record a FMSS (Magaña et al., 1986) which was subsequently coded using the “Criticism” and “Warmth” subscales of the Family Affective Attitude Rating Scale (FAARS; Bullock et al., 2005). The content and tone of the entire speech sample was rated on 12 items assessing parental attributions of their child’s behavior, personality traits, and the parent-child relationship (see Table 1). Each item was rated on a 9-point scale: 1 (*no evidence of the item being coded*) to 9 (*two or more concrete examples*). The total Warmth scale includes 6 items (subcomponents tested in analyses are italicized): positive regarding behavior of child; *positive regarding traits/personality of child*; reports positive relationship with child; *assumes/attributes positive intentions of child;* reports engaging in shared activities with child; and *statements of love/caring toward child*. The total Criticism scale includes 6 items (subcomponents tested in analyses are italicized): critical regarding behavior of child; *critical of traits/personality of child*; *negative relationship with child including signs of anger, resentment, and/or contempt*; negative humor/sarcasm regarding child; *assumes/attributes negative intentions of child;* reports of conflict with anger/hostility toward child). The specific Warmth and Criticism subcomponents chosen for further analysis were those which (1) best mapped onto previous research examining parental attributions of their children with elevated CU traits, and (2) tapped into cognitions surrounding parents’ feelings towards their child with respect to feelings of warmth/positivity versus harshness/criticism. The current study found FAARS intraclass correlation coefficient scores to range from acceptable to excellent for the Warmth dimensions (0.71 – 0.97), and acceptable to very good for the Criticism dimensions (0.57 – 0.86).

**Table 1:** Parenting Dimension, Measure, and Subcomponents in the Present Study

Parenting Dimension	Measure	Sub/Components (Examples)
Warm, Positive Parenting		
Expressed Parental Warmth	FMSS	- Total warmth score - Positive regarding child’s personality traits (e.g., “She is a really good student”) - Assumes/attributes positive intentions of child (e.g., “He keeps the house tidy because he is trying to make my life easier”) - Statements of love/caring toward child (e.g., “I love Tom”)
Observed Positive Parenting Behaviors	DPICS “Do” skills	- Total positive parenting - Labeled praise (e.g., “I love how gently you are building the tower”) - Unlabeled praise (e.g., “Great job”) - Behavior descriptions (e.g., “You’re putting the blue block on top of the red block”) - Reflections (e.g., Child: “I want to build it really high”, Parent: “You want to build it really high”)
Harsh, Negative Parenting		
Expressed Parental Criticism	FMSS	- Total criticism score - Critical of child’s personality traits e.g., (“He’s so selfish”) - Assumes/attributes negative intentions of child (e.g., “She picks on her little sister because she’s jealous of her”) - Negative relationship with child (including signs of anger, resentment, contempt, e.g., “Sometimes I just feel like hitting him”)
Observed Negative Parenting Behaviors	DPICS “Don’t” skills	- Total negative parenting - Questions (e.g., “What do you want to do?”) - Negative statements (e.g., “That’s not the right color”) - Direct and indirect commands (during child-led play, e.g., “Come here” or “Put this piece on next, okay?”

*Note*. DPICS = Dyadic Parent-Child Interaction Coding System, fourth edition; FMSS = five-minute speech sample

#### Observed Positive and Negative Parenting

Video recordings of parent-child interactions during three, standard five-minute observational interaction tasks (i.e., low demand child free-play, medium demand parent-led play, high demand clean-up) completed with one parent at a time were coded by trained independent coders (*k* = 14) using the Dyadic Parent-Child Interaction Coding System, fourth edition (DPICS-IV; Eyberg et al., 2013). The observational parent-child tasks implemented in the present study (i.e., child-led play, parent-led play, and clean-up) are standardized, evidence-based scenarios created specifically for the population of children aged 2–7 years with disruptive behavior problems (Eyberg & Funderburk, 2011; Cotter & Brestan-Knight, 2020). Positive parenting behaviors (i.e., “Do” subcomponent skills of behavior descriptions, reflections, and unlabeled and labeled praises) and negative parenting behaviors (i.e., “Don’t” subcomponent skills of questions and negative talk across all three scenarios, plus indirect and direct commands occurring during the child-led play scenario) were summed across the three standardized situations to compute a single composite score for total positive and negative parenting behaviors (Table 1; Danko et al., 2016).

### Planned Analyses

To test our hypotheses regarding whether parents of children categorized into CP subtypes on the basis of CU traits and internalizing scores differ on warm/positive parenting and harsh/negative parenting and specific subcomponents of these parenting dimensions, we examined ANCOVAs with Bonferroni-corrected planned comparisons between the three groups: CP-only, primary CU variant, and secondary CU variant. For all comparisons, child CP severity and age were entered as covariates to ensure that differences in parenting were not due to the greater severity of CP in the CU groups or to changes over time in parenting a child with CP. Parents’ total number of verbalizations was also entered as a covariate for analyses examining observed DPICS positive and negative parenting behaviors to account for overall engagement and talkativeness of the caregiver. All tests were run separately for mothers and fathers, however, due to the small sample size within each CP subtype when using father-reported data (CP-only: *n* = 17; primary CU variant: *n* = 12; secondary CU variant: *n* = 9), only mother-reported data were subsequently analyzed and reported. See Supplemental Table 1 for results of planned comparisons using father-reported data. We report 95% simultaneous confidence intervals (CIs), and partial eta-squared (η_p_^2^) and Cohen’s *d* effect size coefficients where relevant (small: η_p_^2^ = 0.01; medium: η_p_^2^ = 0.06; large: η_p_^2^ = 0.14; Cohen, 1988).

## Results

### Correlations Between Main Study Variables

Data were screened for outliers and assumptions tested (see Supplemental Statistical Materials for screening procedure). Table 2 presents bivariate Pearson’s zero-order correlations between main study variables. Partial correlations controlling for CP severity and parent verbosity are presented in parentheses. SS-warmth and SS-criticism were significantly negatively associated. DPICS positive and negative parenting behaviors were significantly positively correlated when running bivariate correlations, and significantly negatively correlated when controlling for children’s level of CP and mothers’ total number of verbalizations during the interaction task. SS-criticism scores were positively associated with both DPICS positive and negative parenting. SS-warmth scores were negatively associated with DPICS positive parenting but uncorrelated with DPICS negative parenting. SS-warmth scores were significantly negatively associated with ICU scores. SS-criticism scores were significantly positively associated with ECBI and internalizing scores. DPICS negative parenting behaviors were non-significantly negatively associated with internalizing scores. ECBI, ICU, and internalizing scores were all significantly positively correlated.

**Table 2:** *Descriptives and Correlations Among Main Study Variables*

Study Variables	1.	2.	3.	4.	5.	6.	7.
1. SS-warmth	1						
2. SS-criticism	− 0.33** (− 0.30**)	1					
3. DPICS positive parenting	− 0.12 [− 0.13]	0.23* [0.24*]	1				
4. DPICS negative parenting	− 0.01 [0.06]	0.13 [0.05]	0.48** [− 0.33**]	1			
5. ECBI	− 0.17	0.25**	0.07 [− 0.06]	0.16 [0.04]	1		
6. ICU	− 0.18* (− 0.12)	0.08 (− 0.03)	− 0.13 [− 0.20*]	0.07 [0.11]	0.44**	1	
7. CBCL INT	− 0.12 (− 0.06)	0.18* (0.08)	− 0.07 [0.05]	− 0.10 [− 0.01]	0.42**	0.37** (0.23**)	1
Mean	4.65	3.69	17.45	49.94	156.93	28.85	60.89
SD	1.53	1.39	11.80	26.16	31.98	9.24	9.43
Skewness	0.23	0.32	0.74	0.12	− 0.58	− 0.30	− 0.38
Kurtosis	− 0.54	− 0.28	− 0.21	− 0.94	0.39	− 0.16	− 0.28

*Note*. Data are presented from the sample of *N* = 135 mothers who completed all expressed parenting measures and questionnaire measures, and the subgroup of *n* = 105 who also completed the DPICS measure. SS = speech sample; DPICS = Dyadic Parent-Child Interaction Coding System; ECBI = Eyberg Child Behavior Inventory; ICU = Inventory of Callous-Unemotional Traits; CBCL = Child Behavior Checklist; INT = Internalizing. Values in round parentheses represent partial correlations controlling for child conduct problems. Values in square brackets represent partial correlations controlling for child conduct problems (except for ECBI scores) and parent verbosity

*p < .05. **p < .01

### Group Differences in Warm/Positive and Harsh/Negative Parenting

Table 3 presents results of the ANCOVA omnibus tests for planned comparisons, which revealed that total SS-warmth was significantly lower for secondary CU variants relative to the CP-only group, *M*diff = -0.92, *p* = .028, 95% CI [-1.76, -0.07], η_p_^2^ = 0.05, *d* = 0.62 but not relative to primary CU variants, *M*diff = -0.48, *p* = .538, 95% CI [-1.34, 0.38], η_p_^2^ = 0.01, *d* = 0.31. Mothers of primary CU variants, *M*diff = -1.09, *p* = .024, 95% CI [-2.07, -0.11], η_p_^2^ = 0.05, *d* = 0.61 attributed significantly fewer positive intentions to their child’s behavior compared to the CP-only group, with no difference between CU variant groups. Planned comparisons did not reveal any significant group differences in mother-reported positive traits or feelings of love/caring for their children. There were no significant group differences in global or subcomponent (i.e., labeled praises, behavior descriptions, reflections) scores for positive parenting.

**Table 3:** *Means (SE) for CP Subtypes on Mothers’ Expressed Warmth and Criticism Total and Subscale Scores, and Observed Positive and Negative Parenting Behaviors*

Expressed Outcomes	CP only (*n* = 59)	Primary (*n* = 38)	Secondary (*n* = 38)	*F* value	*p-*value	*df*	η_p_^2^	*d*
Total Warmth	5.04^a^ (0.20)	4.60^ab^ (0.24)	4.12^b^ (0.26)	3.51	0.033	2	0.05	0.59
Positive Traits	8.57 (0.27)	8.55 (0.32)	7.51 (0.34)	3.18	0.045	2	0.05	0.54
Positive Intent	3.33^a^ (0.26)	2.24^b^ (0.31)	2.45^ab^ (0.33)	4.05	0.020	2	0.06	0.67
Love/Caring	3.51 (0.35)	3.38 (0.42)	2.76 (0.45)	0.83	0.438	2	0.01	0.46
Total Criticism	3.66 (0.19)	3.49 (0.22)	3.93 (0.24)	0.97	0.380	2	0.02	0.59
Critical Traits	6.11 (0.39)	6.07 (0.47)	6.85 (0.50)	0.80	0.453	2	0.01	0.55
Negative Intent	1.78 (0.30)	2.71 (0.35)	2.27 (0.38)	2.02	0.137	2	0.03	0.45
Negative Relationship	3.58^ab^ (0.37)	2.69^a^ (0.44)	4.30^b^ (0.47)	3.28	0.041	2	0.05	0.81
Observed Outcomes	CP only (*n* = 48)	Primary (*n* = 27)	Secondary (*n* = 30)	*F* value	*p-*value	*df*	η_p_^2^	*d*
Positive Parenting Behaviors	19.11 (1.28)	14.55 (1.63)	17.42 (1.67)	2.41	0.095	2	0.05	2.07
Behavior Descriptions	1.26 (0.38)	0.86 (0.48)	0.67 (0.49)	0.46	0.633	2	0.01	0.35
Reflections	7.34 (0.81)	5.98 (1.04)	7.70 (1.06)	0.81	0.449	2	0.02	1.30
Labeled Praise	1.26 (0.25)	1.28 (0.32)	1.53 (0.33)	0.21	0.811	2	0.00	1.15
Unlabeled Praise	9.56 (0.88)	6.97 (1.12)	8.84 (1.15)	1.69	0.191	2	0.03	1.67
Negative Parenting Behaviors	49.32 (2.03)	51.70 (2.59)	49.37 (2.65)	0.31	0.735	2	0.01	3.48
Questions	39.15 (1.82)	40.67 (2.31)	37.50 (2.36)	0.46	0.632	2	0.01	3.35
Negative Talk	5.73 (0.92)	5.06 (1.17)	4.75 (1.20)	0.21	0.809	2	0.00	0.85
Commands During CDI	4.53 (0.97)	5.99 (1.22)	7.12 (1.25)	1.24	0.295	2	0.03	0.70

*Note*. Estimated marginal means (SE); different superscripts (^a, b^) denote significant differences between groups in pair-wise comparisons. Primary = high CU traits and low INT; Secondary = high CU traits and high INT; CDI = child-directed interaction. *F*-value, *p*-value, *df*, η_p_^2^, and Cohen’s *d* represent statistics for omnibus tests of group differences. CP severity and child age entered as covariates for all analyses. Total number of mother verbalizations additionally entered as covariate only for positive and negative DPICS parenting behaviors analyses

Planned comparisons did not indicate any significant group differences in mothers’ total expressed criticism, in negative reports of their child’s dispositional traits, or in mothers’ attributions of negative intentions to their child’s behavior. However, mothers expressed having more negative relationships with secondary CU variants compared to primary CU variants, *M*diff = 1.61, *p* = .038, 95% CI [0.07, 3.15], η_p_^2^ = 0.05, *d* = 0.76, but did not differ from the CP-only group *M*diff = 0.72, *p* = .753, 95% CI [-0.80, 2.24], η_p_^2^ = 0.01, *d* = 0.53. There were no significant differences between primary CU and CP-only groups, *M*diff = -0.89, *p* = .370, 95% CI [-2.27, 0.50], η_p_^2^ = 0.02, *d* = 0.20. Planned comparisons did not reveal any significant group differences in overall observed negative parenting or its subcomponents (i.e., negative talk, asking questions, or issuing commands during free-play). Since the majority of prior psychometric and construct validity research on CU traits has been conducted with children ages 3 and older, and to address concerns around the validity of measuring CU traits in very young children (Waller et al., 2016), we re-ran analyses excluding 2-year-old children (*n* = 20) (See Supplemental Table 2).

## Discussion

The present study sought to clarify the role of warm/positive and harsh/negative dimensions of parenting factors and their subcomponents in childhood CP subtypes, differentiated on the basis of CU traits and internalizing symptomatology. Key strengths of this study were the multi-method approach used to assess parenting dimensions in a clinic-referred sample of children and our inclusion of both global dimensions of expressed and observed parental warmth and criticism, and specific, empirically-supported subcomponents of these constructs. This demarcation allowed us to demonstrate that a global measurement approach may fail to capture nuanced differences in how subcomponents of broader dimensions of parenting factors operate for each CP subtype/CU variant. Support for our hypotheses regarding the global dimensions of expressed and observed parental warmth/positivity and harshness/negativity was mixed: As predicted, mothers of children classified as secondary CU variants expressed significantly less global/total warmth than mothers of children in the CP-only group. However, CU variants did not differ on either total expressed warmth or the composite of observed positive parenting behaviors. Also contrary to hypotheses, CP subtypes did not differ on total expressed criticism or observed negative parenting behaviors. When subcomponents of these global dimensions were considered, in partial support of hypotheses, we found that mothers of children classified as secondary CU variants were significantly more likely than mothers of children with primary CU traits to endorse a negative relationship with their child. Furthermore, mothers of primary CU variants were significantly less likely to ascribe positive intentions to their child’s behaviors toward others than mothers of CP-only children. We discuss four key findings in more detail.

First, this is the first study to demonstrate that specific aspects of expressed maternal warmth and criticism differentiate young children classified into CP subtypes/CU variants. Mothers of children with secondary CU traits were more likely to endorse a negative relationship with their child than mothers of primary CU variants. As expressed by mothers, these negative parent-child relationships were characterized by feelings of anger, resentment, hostility, contempt, and active avoidance of and/or desire to harm the child. This finding is consistent with broader theory and research regarding the etiology of secondary CU variants as characterized by experiences of adverse and traumatic early-life events, including abusive home environments and maltreatment (Craig et al., 2021b; Goulter et al., 2017). This finding is also consistent with prior research in adolescents with secondary CU traits, who self-reported significantly higher levels of parental hostility compared to adolescents with primary CU traits (Flexon, 2015). However, our findings are inconsistent with prior research with young children that found no evidence for variant differences in parental negative feelings or harshness (Ezpeleta et al., 2017; Humayun et al., 2014; Meehan et al., 2017). This inconsistency underscores the importance of study replication using clinic-referred samples and methodological paradigms beyond parent-report questionnaires that are subject to bias. Our findings of the relative importance of maternal expressed warmth and its subcomponent tapping into the parent’s feelings towards their child for the secondary CU group is consistent with a recent study that identified parental warmth as a predictor of later membership into the secondary, but not primary, CU variant group (Craig et al., 2021a).

These findings add to evidence supporting CU variant differences in parenting factors that are identifiable early in child development, especially as they relate to mothers’ cognitions and feelings about the mother-child relationship. Yet it remains unclear why mothers of children with secondary CU traits are particularly hostile in their cognitions, and what child and/or parent factors are at play in this parent-child dynamic. Since negative cognitions regarding the parent-child relationship are likely to contribute to consistently higher rates of abuse and neglect reported among older youth with secondary CU traits (Craig et al., 2021b), our findings encourage further study regarding the source of the harmful maternal cognitions specifically for this CP subtype.

However, not all subcomponents of expressed warmth and criticism were worse for children in the secondary CU group. Indeed, our second key finding is that mothers of children classified with primary CU traits expressed fewer positive attributions about their child’s intentions towards others than children with CP-only. This finding indicates that mothers of children with elevated CU traits and low levels of anxiety tended to assume their child’s behavior was driven by fewer altruistic intentions and were less likely to perceive an objectively positive behavior (e.g., tidying bedroom) as being helpful or beneficial except to the child. From a cognitive-behavioral perspective, negative cognitions regarding the intentions driving child behavior are likely to influence parent behavior in response to the child; for example, by undermining parent recognition and praise for helpful behaviors. Thus, mothers’ negative cognitions regarding a child’s behavioral intentions may serve to maintain behavior problems specifically among children with elevated CU traits.

Our third key finding however, is that for the present study, the CP-only and primary CU groups did not differ on most other parenting measures examined. This is unsurprising, as past literature has presented a somewhat mixed view on parenting practices relevant for the CP subtypes. The literature commonly links harsh parenting with CP-only and secondary CU variants, and low parental expressed and observed warmth with the primary CU variant (Goulter et al., 2017; Craig et al., 2021a; Ezpeleta et al., 2017; Viding et al., 2009; Pasalich et al., 2011a). We offer two possible explanations for why our primary CU and CP-only groups did not significantly differ across most parenting measures. Firstly, children with elevated CU traits exhibit earlier onset and more severe CP than children with low levels of CU traits (Frick et al., 2014). Hence, the CP-only group in the present study may have been somewhat elevated on CU traits, as this sample of children was clinic-referred for CP. A second possibility is that the CP of the young primary CU children in the current study are not yet as severe as is typically seen in older populations, with parental attributions between CP-only and primary CU groups being mostly indistinguishable until the CP and CU behaviors of the latter increase over time. This hypothesis is supported by Ezpeleta et al. (2017) who identified a group of primary CU variants who showed increasing levels of CP between ages 3 and 7 years. If this young age range signals the start of a process that leads to the pronounced group differences in CP and CU commonly seen in the CU literature, then this would further support the need for targeted early intervention for young children with elevated CU traits.

Nevertheless, when considered together, our first and second key findings highlight the importance of considering the specific nature of parental cognitions about the child’s personality and behavior and the parent-child relationship. Consistent with prior literature with older samples (Bégin et al., 2021), children with elevated CU traits – irrespective of variant – received less warm and positive and more harsh and negative attributions than their CP-only counterparts; however, the specific nature of mothers’ expressed parenting differed between the variants. Drawing together the findings from the current study, secondary CU traits were cross-sectionally associated with parenting that is less warm and more harsh than other CP subtypes, but this association seems to be driven by mothers’ cognitions about the child in the context of the parent-child relationship. In contrast, primary CU variants appear to be associated with less warm parenting driven by mothers’ cognitions about what the child *does* and the intention/why these behaviors occur. In both cases, the cognitions are ‘child-referent’ rather than ‘parent-referent’, which influences the extent to which parents perceive child problems as stable, permanent, and intentional – dimensions known to elicit negative affective and behavioral responses from parents following disruptive behavior (Morrissey-Kane & Prinz, 1999). It is thus unsurprising that parent attributions have been identified as both a treatment target and moderator of treatment response to interventions for young children with CP generally (see Sawrikar and Dadds, 2018 for a review). However, existing research supports a positive association between child CU traits and negative parental attributions, above and beyond other child and parent factors (Palm et al., 2019; Sawrikar et al., 2019). This may help explain why young children with CP and CU traits typically receive less benefit from traditional interventions for CP (Wilkinson et al., 2016), since parent training protocols rarely directly target parent cognitions (Sawrikar & Dadds, 2018). Accordingly, the efficacy of programs specifically developed for children with CU traits (e.g., PCIT-CU) is likely to be improved by including specific parent attributions as a key treatment target (Fleming et al., 2022), which may need to differ based on CU variant classification.

The fourth and final key finding of the current study was that CP subtypes did not differ on the observed parenting measure. This was unexpected given previous research demonstrating that parents who were physically abusive responded more negatively to their children and were more controlling during free-play than non-abusive parents (Borrego et al., 2004), particularly since abuse has been linked to the CP of children with CP-only and secondary CU traits. The significant group differences identified for the expressed parenting measures, but not the observed parenting measures suggests a disconnect: At least within the research clinic observation context, mothers of children with elevated CU traits are not displaying more negative (e.g., verbal criticism) or less positive (e.g., labeled praise) parenting behaviors than mothers of children with CP-only, despite thinking and feeling more negatively and less positively about their child. This disconnect between parents’ expressed and observed parenting may account for some of the prior inconsistencies on the roles of warm/positive and harsh/negative parenting factors between CU variants (Ezpeleta et al., 2017; Craig et al., 2021a; Goulter et al., 2017), which diverge in how they operationalize and measure parenting. This finding raises the possibility that the lack of differences for the observed parenting measure in the present study was due to impression management, where parents may attempt to create a positive impression of their parenting/the parent-child relationship, since they were aware of being observed and videotaped. Moreover, all parents were made aware of the ‘mandatory reporter’ status of assessors at the outset of the baseline assessment during which parent-child observations were conducted, incentivizing parents to avoid behaviors that may lead to referral to relevant child protective services (e.g., emotional abuse, corporal punishment). In contrast, parents completed the FMSS procedure alone, possibly providing a sense of privacy, and fewer cues to monitor and modify behavior. Future research may benefit from examining observed parenting behaviors in a more naturalistic setting to increase the ecological validity of this measure and help elucidate differences in parenting behaviors between CP subtypes.

An unexpected finding from our study was that ICU and ECBI scores were only weakly to moderately correlated with many of our parenting measures. All children in the current study had clinically significant CP, which may have limited the variability in ECBI scores to detect associations with parenting factors. However, the literature is also mixed regarding the relevant parenting factors for CP subtypes. Some studies find evidence for a moderating role of CU traits on the relationship between CP and harsh parenting (Wootton et al., 1997; Pasalich et al., 2011a), whereas others do not (Waller et al., 2015a). Other research suggests that harsh parenting is more relevant for children with CP-only and secondary variants (Goulter et al., 2017; Viding et al., 2009) than primary variants. However, the present study oversampled children with CU traits, which allowed us to capture the less prevalent secondary CU variants. We found that the parents of these secondary CU variants expressed significantly more harsh parenting attributions and less warm attributions relative to other clinical conduct groups. Combining these three CP subgroups into one group would have washed out the significant associations between CP and parenting factors found in the present study. Hence, our finding of non-significant correlations between ICU and ECBI scores with many parenting measures thus supports the importance of disaggregating children with CP into subgroups based on CU traits and internalizing problem scores, since aggregating them risks obfuscating real group differences.

The current study had some limitations. First, despite attempts to engage all primary caregivers, few fathers participated in this research, and there was subsequently low power to detect group differences using father-reported measures. Second, we did not include a measure of child trauma and/or maltreatment in this study, preventing us from comparing CU variants on their levels of exposure to these traumatic early-life experiences. Lastly, we did not include a ‘low problems’ or ‘typically-developing’ control group or an internalizing-only clinical group in this study, limiting our capacity to compare patterns of parenting factors among clinical and non-clinical populations. On the other hand, this was the first study to compare CU variants to a CP-only group in a sample of clinic-referred young children with CP, indicating that the results of this study may be generalizable to other clinical samples of children with CP. Given the well-characterized clinical groups in this study, and the restricted range in CU traits for this clinical sample of children, the current study took a group-based median-split approach to investigating CU variants. An important future research direction is to establish cut-off scores on the ICU in young child populations, to assist in classifying children with CP into subtypes on the basis of elevated CU traits. Future research with samples with more variable CU trait scores may also consider taking a dimensional approach to investigating parenting factors for differentiating CP subtypes, as the present study relied upon a median-split approach which is sample dependent. An important future research direction is to establish cut-off scores on the ICU in preschool populations, to assist in classifying children with CP into subtypes on the basis of elevated CU traits.

This study has several key strengths. Beyond being the first study to investigate multidimensional parenting factors differentiating CP subtypes in a sample of clinic-referred young children, it also used a multi-method approach by incorporating both expressed and observed measures of positive and negative parenting, as well as examining relevant subcomponents of expressed and observed parental warmth and criticism. We found that mothers of children with secondary CU traits reported having a more negative parent-child relationship relative to mothers of children with primary CU traits. Also, mothers of children with primary CU traits ascribed fewer positive intentions to their children’s behaviors, relative to the CP-only group. Understanding how parenting factors differ between CP subtypes is vital for informing intervention efforts for this population of children. In particular, the findings of this study highlight the need to tailor treatment to match the needs of the family, based on the identified presence of CP, CU traits and/or internalizing symptomatology. Tailored treatment approaches for children on different developmental trajectories are vital for enhancing treatment efficacy and efficiency.

### Electronic Supplementary Material

Below is the link to the electronic supplementary material.

Supplementary Material 1

Supplementary Material 2
