# Supplementary material for: Dimensions of Warm Parenting Attributions Differentiate Conduct Problem Subtypes in Young Children
Source: Res Child Adolesc Psychopathol. 2023 Aug 15;52(2):223–36. doi: 10.1007/s10802-023-01111-7 (PMC10834570; doi:10.1007/s10802-023-01111-7)
Supplement: Supplementary file 1 — Supplementary Material 1 [file 10802_2023_1111_MOESM1_ESM.pdf]

Supplemental Methodological Materials

The following provides details regarding the administration, scoring, and established psychometric properties of each study measure.

Measures

***Conduct Problems*.** Mothers rated the frequency of the child's problem behaviors (e.g., "has temper tantrums") on a 7-point scale, from 1 (*never*) to 7 (*always*), with scores summed to compute total Intensity Scale scores. Intensity Scale scores range between 36-252 and demonstrated excellent internal consistency (Cronbach's α = 0.95; Eyberg and Pincus, 1999), inter-parent reliability (α = 0.69; Eisenstadt et al., 1994), and test-retest reliability across 12 weeks and 10 months (α = 0.80 and 0.75, respectively; Funderburk et al., 2003).

***CU Traits*.** Mothers rated the applicability of each item on a 4-point scale from 0 (*not at all true*) to 3 (*definitely true*), with total scores ranging from 0-72. For the present study, ICU total scores demonstrated acceptable internal consistency and expected correlations with criterion measures such as reduced emotional responding to distress cues and severe aggression, across a wide age range, sex, types of samples, and different language translations (e.g., Ezpeleta et al., 2013; Kimonis et al., 2016). Preschool children rated high on the ICU by parents and teachers were more likely to be antisocial and aggressive, score high on other psychopathy dimensions, and show emotion recognition deficits than children scoring low (Kimonis et al., 2016).

***Internalizing Symptoms*.** Mothers rated the applicability of each item on a 3-point scale from 0 (*not true*) to 2 (*very true or often true*), with scores summed to compute the composite score. The Internalizing composite scale of the ASBEA demonstrated excellent internal consistency and test re-test reliability (Achenbach & Rescorla, 2001).

Expressed Parental Criticism and Warmth. Scores obtained using the FAARS coding scheme have good internal reliability and convergent validity with other self-reported and observed parenting measures (Waller et al., 2012; Pasalich et al., 2011b). Codes were deemed reliable once trained coders (k = 2) achieved 80% agreement with the expert coder on criterion videos, before coding for this study commenced (Waller et al., 2015a).

Observed Positive and Negative Parenting. DPICS-IV scores show adequate to strong inter-rater reliability and significant convergent validity with other measures of parenting (Eyberg et al., 2013). Scores also show the ability to screen clinical from non-clinical samples of 2-7-year-old children with and without CP (Bjørseth et al., 2015). For the present study, coders were trained to reliability by an expert DPICS coder and deemed reliable once they achieved 80% agreement with the expert coder on criterion videos.
