# Supplementary material for: Dimensions of Warm Parenting Attributions Differentiate Conduct Problem Subtypes in Young Children
Source: Res Child Adolesc Psychopathol. 2023 Aug 15;52(2):223–36. doi: 10.1007/s10802-023-01111-7 (PMC10834570; doi:10.1007/s10802-023-01111-7)
Supplement: Supplementary file 2 — Supplementary Material 2 [file 10802_2023_1111_MOESM2_ESM.pdf]

Supplemental Statistical Materials

Data Screening Procedure

Prior to conducting main study analyses, data were screened for outliers and assumptions of planned analyses were tested. Outliers for observational measures were identified by a z-score of ±3 and were 90% winsorized, with scores below/above the 5th/95th percentile truncated to the 5th/95th percentile (Aguinis et al., 2013). Using this method, two data points for DPICS positive parenting scores were winsorized.

To test for normality in our data, we examined the skewness and kurtosis of residual distributions. None of the observational measures were skewed or kurtotic. Parent-reported measures that were skewed and/or kurtotic were retained in their raw form as these scores reflect true parent-reported values and as such were not winsorized or transformed.

Analytic Procedure

Independent samples t-tests were conducted to compare younger (2-4 years) versus older children (5-7 years) on main study variables, to assess for potential differences between preschool-age versus school-age children. Internalizing (INT) scores were significantly higher for younger children (M = 62.35, SD = 9.28) than older children (M = 56.19, SD = 8.44), t(133) = 3.35, p = .001, 95% CI [2.52, 9.80]. Mothers of younger children expressed significantly more statements of love and care toward their child (M = 3.52, SD = 2.71) than mothers of older children (M = 2.41, SD = 1.98), t(133) = 2.16, p = .033, 95% CI [0.09, 2.14]. Mothers also demonstrated significantly higher DPICS negative parenting scores for older children (M = 62.20, SD = 25.67) than younger children (M = 46.11, SD = 25.27), t(103) = -2.77, p = .007, 95% CI [-27.61, -4.56], including asking more questions during play for older children (M = 50.88, SD = 21.40) than younger children (M = 35.38, SD = 21.92), t(103) = -3.10, p = .002, 95% CI [-25.41, -5.60].

Overall, mothers demonstrated significantly less DPICS positive parenting scores for younger children (*M* = 16.19, *SD* = 11.45) than older children (*M* = 21.50, *SD* = 12.24), *t*(103) = -1.99, *p* = .049, 95% CI [-10.60, -0.03]. Mothers also tended to make more verbalizations during play with their children who were older (*M* = 169.64, *SD* = 53.08) versus younger (*M* = 125.56, *SD* = 60.01), *t*(103) = -3.29, *p* = .001, 95% CI [-70.65, -17.51]. This included less labeled and unlabeled praise for younger children (*M* = 1.10, *SD* = 1.63; *M* = 7.88, *SD* = 6.80) than older children (*M* = 2.12, *SD* = 2.40; *M* = 11.28, *SD* = 8.62), *t*(103) = -2.42, *p* = .017, 95% CI [-1.86, -0.18] and *t*(103) = -2.05, *p* = .043, 95% CI [-6.71, -0.10], respectively.

We also conducted independent samples t-tests to compare boys and girls on main study variables, although found no significant group differences. Main study analyses were repeated controlling for child age, sex, and clinic site, although results did not differ when including sex or clinic site as a covariate (results available upon request). Hence, only child age was included as an additional covariate, to allow for the most parsimonious account of the data.

Within our original sample, there was no difference in the sex, CP, CU traits, or INT of children whose mothers had completed all study measures compared with mothers with incomplete measures. However, children whose mothers had not completed all study measures were significantly younger (*M* = 3.65, *SD* = 1.29) than mothers who had completed all study measures (*M* = 4.15, *SD* = 1.25), *t*(195) = -2.77, *p* = .006, 95% CI [-0.86, -0.14]. Within our final sample of mothers used for the present study (*N* = 135), there was no significant difference in the sex, age, CP, CU traits, or INT of children with complete versus incomplete DPICS behavioral observations.

Supplemental Table 1

Means (SE) for CP subtypes on Fathers' Expressed Warmth and Criticism Total and Subscale Scores.

Expressed Outcomes	CP-only (n = 17)	Primary (n = 12)	Secondary (n = 9)	F value	p-value	df	η_p^2	d
Total Warmth	4.50 (0.29)	4.62 (0.34)	3.76 (0.40)	1.49	.241	2	0.08	1.30
Positive Traits	7.68[a] (0.59)	7.20[ab] (0.69)	5.01[b] (0.83)	3.38	.046	2	0.17	1.16
Positive Intent	2.59 (0.36)	2.38 (0.43)	1.83 (0.51)	0.71	.500	2	0.04	0.82
Love/ Caring	3.28 (0.59)	3.63 (0.69)	4.19 (0.83)	0.38	.687	2	0.02	0.75
Total Criticism	3.52 (0.31)	2.92 (0.37)	2.58 (0.44)	1.65	.209	2	0.09	0.92
Critical Traits	6.17 (0.77)	5.01 (0.90)	3.67 (1.08)	1.72	.195	2	0.09	0.84
Negative Intent	2.23 (0.54)	2.54 (0.63)	1.52 (0.76)	0.54	.589	2	0.03	0.46
Negative Rel	2.84 (0.61)	2.63 (0.72)	3.25 (0.86)	0.15	.858	2	0.01	0.63

Note. Estimated marginal means (SE); different superscripts ([a, b]) denote significant differences between groups in pair-wise comparisons. Primary = high CU traits and low INT; Secondary = high CU traits and high INT. F-value, p-value, df, and η_p^2 represent statistics for the omnibus tests of group differences. CP severity and child age entered as covariates for all analyses.

Supplemental Table 2

Means (SE) for CP Subtypes on Mothers' Expressed Warmth and Criticism Total and Subscale Scores, and Observed Positive and Negative Parenting Behaviors, Excluding 2-Year-Old Children.

Expressed Outcomes	CP only (*n* = 57)	Primary (*n* = 28)	Secondary (*n* = 30)	*F* value	*p*-value	*df*	η_p^2	*d*
Total Warmth	4.74 (0.21)	4.70 (0.28)	4.22 (0.29)	1.04	.358	2	0.02	0.49
Positive Traits	8.49 (0.27)	8.51 (0.36)	7.94 (0.37)	0.82	.445	2	0.02	0.38
Positive Intent	3.30[a] (0.26)	1.97[b] (0.35)	2.23[ab] (0.36)	5.26	.007	2	0.09	0.75
Love/ Caring	3.10 (0.36)	3.62 (0.49)	2.83 (0.50)	0.71	.492	2	0.01	0.42
Total Criticism	3.87 (0.19)	3.52 (0.25)	3.72 (0.26)	0.60	.550	2	0.01	0.59
Critical Traits	6.32 (0.42)	5.93 (0.57)	6.57 (0.59)	0.34	.715	2	0.01	0.57
Negative Intent	1.99 (0.33)	2.85 (0.44)	2.29 (0.46)	1.19	.309	2	0.02	0.38
Negative Relationship	3.80 (0.37)	2.67 (0.50)	3.92 (0.52)	2.13	.124	2	0.04	0.83
**Observed Outcomes**	**CP only (*n* = 48)**	**Primary (*n* = 20)**	**Secondary (*n* = 23)**	***F* value**	***p*-value**	***df***	η_p^2	***d***
Positive Parenting Behaviors	19.66[a] (1.31)	13.49[b] (1.91)	17.87[ab] (1.94)	3.51	.034	2	0.08	2.01
Behavior Descriptions	1.24 (0.40)	0.95 (0.59)	0.51 (0.60)	0.46	.636	2	0.01	0.38
Reflections	7.22 (0.84)	5.90 (1.23)	8.42 (1.25)	1.07	.346	2	0.03	1.25

Labeled Praise	1.50 (0.27)	1.06 (0.39)	1.60 (0.40)	0.59	.559	2	0.01	1.14
Unlabeled Praise	10.08 (0.93)	6.33 (1.36)	8.95 (1.38)	2.55	.084	2	0.06	1.60
Negative Parenting Behaviors	51.40 (2.04)	55.43 (2.98)	50.27 (3.03)	0.90	.409	2	0.02	3.36
Questions	41.22 (1.81)	44.61 (2.66)	37.61 (2.70)	1.76	.179	2	0.04	3.30
Negative Talk	5.94 (0.89)	4.58 (1.30)	5.15 (1.32)	0.38	.687	2	0.01	0.79
Commands During CDI	4.34 (0.99)	6.26 (1.43)	7.48 (1.46)	1.52	.225	2	0.04	0.73

Note. Results in this table reflect a subsample of mothers with children aged 3-years-old and above (i.e., all 2-year-old children have been removed from these analyses). Estimated marginal means (SE); different superscripts ($^{a, b}$) denote significant differences between groups in pair-wise comparisons, following Bonferroni adjustment. Primary = high CU traits and low INT; Secondary = high CU traits and high INT; CDI = child-directed interaction. *F*-value, *p*-value, *df*, η_p^2, and Cohen's *d* represent statistics for omnibus tests of group differences. CP severity and child age entered as covariates for all analyses. Total number of mother verbalizations additionally entered as covariate only for positive and negative DPICS parenting behaviors analyses.
